# Gender and immunosuppression impact on Merkel cell carcinoma diagnosis and prognosis. A population based cohort study

**DOI:** 10.1002/ski2.80

**Published:** 2021-12-08

**Authors:** E. Keeling, E. O'Leary, S. Deady, J. P. O Neill, P. J. Conlon, F. J. Moloney

**Affiliations:** ^1^ Department of Dermatology Tallaght University Hospital Dublin Ireland; ^2^ National Cancer Registry Cork Ireland; ^3^ Otolaryngology Department Beaumont Hospital Dublin Ireland; ^4^ Royal College of Surgeons in Ireland Dublin Ireland; ^5^ Nephrology Department Beaumont Hospital Dublin Ireland; ^6^ Department of Dermatology Mater Misericordiae University Hospital Dublin Ireland; ^7^ School of Medicine University College Dublin Dublin Ireland; ^8^ Melanoma Institute Australia Sydney New South Wales Australia

## Abstract

**Background:**

Merkel cell carcinoma (MCC), a rare cutaneous neuroendocrine endocrine tumour is increasing in incidence, and continues to carry a poor prognosis.

**Objectives:**

The objectives of this study were to examine all Irish cases of MCC from 1 January 1994 over 2 decades, focusing on gender and organ transplantation recipients (OTRs). Cases were identified from the National Cancer Registry of Ireland. Covariates of interest included age, body site, period of diagnosis, deprivation‐status and history of non‐melanoma skin cancer (NMSC).

**Results:**

In total 314 MCC cases were identified. A female predominance was noted (53.8%). Comparison between age‐standardised rates between the earliest period (1994–1996) with the latest period (2012–2014) showed an increase of 105% in total. The trend in age‐standardised incidence rates were noted to be increasing significantly (*p* = 0.0004). Average age at diagnosis was 77.6 years (male 75.1 years, female 79.7 years). Overall, the majority of MCC cases presented on the head and neck (*n* = 170, 54.1%). Differences in anatomical location of MCCs were noted between genders. Males were found to be more likely to have a history of previous NMSCs (males *n* = 73 [57.9%], females *n* = 53 [42.1%]). Thirty‐one percentage of patients died from MCC, average survival was 3.5 years in those who died of this malignancy. Ten organ transplant recipients developed MCC. OTR who developed MCC were diagnosed at a younger average age of 65.1 years. Standardized incidence ratio for MCC in OTR was 59.96. A higher proportion of OTR died from MCC (70%), with a shorter median survival of 0.14 years. In competing risks regression, gender was not significantly associated with risk of dying, females having a non‐significantly higher hazard of dying. Organ transplant recipients and patients from less deprived areas were at greater risk of dying from MCC.

**Conclusions:**

This population based study provides epidemiological, clinical and outcome data for MCC over a 20‐year period.

## Graphical abstract

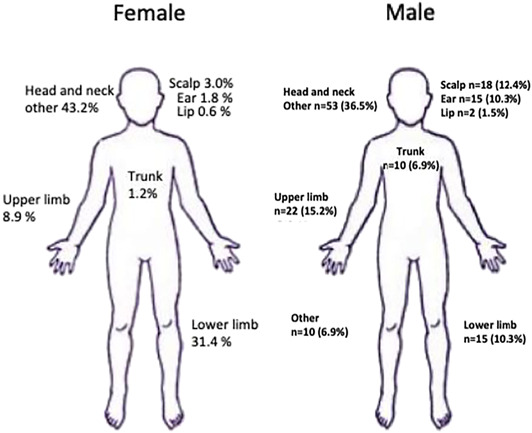

**What's already known about this topic?**

- Merkel cell carcinoma (MCC) is an aggressive cutaneous neuroendocrine skin cancer, most likely arising from basal stem cells that show neuroendocrine differentiation.
- While rare, it is increasing in incidence.
- MCC portends a poor prognosis.
- The pathophysiology is incompletely understood, however photo damage and Merkel cell polyoma virus play a role. Immunosuppressed patients are at increased risk from MCC.

**What does this study add?**

- This study examined MCC in the Irish population over a 20‐year period from 1994 to 2014, identifying 314 cases from the National Cancer Registry.
- Females developed MCC at an older age, on sites with less sun exposure, are less likely to have had prior non‐melanoma skin cancers, and have a longer survival time than their male counterparts.
- In multivariate competing risks survival analysis, organ transplant recipients and patients from less deprived areas were at greater risk of dying from MCC.

## 1 INTRODUCTION

Merkel cell carcinoma (MCC) is an aggressive cutaneous neuroendocrine skin carcinoma, most likely arising from basal stem cells that show neuroendocrine differentiation.^1^ While MCC is rare, with an annual incidence rate of 0.7 per 100 000 persons,^2^ numerous population based studies demonstrate increasing incidence.^2^, ^3^ Previous research has shown MCC has lower cumulative overall average survival rates compared with melanoma from 12 months extending to 10 years post diagnosis.^4^

The pathophysiology of MCC remains incompletely understood. It is recognized that Merkel cell polyoma virus (MCPyV) is integrated into the host genome in a monoclonal pattern in approximately 80% of MCC. In contrast, MCPyV negative tumours demonstrate signature ultraviolet radiation mutations with a much higher mutational burden.^5^ Recent data on survival suggests better outcomes for women than men diagnosed with MCC.^6^ In contrast, there is a marked increased risk of MCC in immunosuppressed individuals, with aggressive tumour behaviour, increased risk of metastases and poorer disease specific survival.^7^

This study examined epidemiological, clinical and outcome data for MCC within the Irish population, including organ transplant recipients (OTRs) over a 20‐year period, focusing on the effect of gender and chronic immunosuppression on diagnosis and prognosis.

## 2 METHODS

All cases of MCC in Ireland are reported to the National Cancer Registry Ireland (NCRI), a national body that collects data on cancer diagnosis and survival in all patients in the Republic of Ireland. It has prospectively collected data since 1 January 1994. Cases of MCC between 1994 and 2014 were identified. Covariates of interest including age, anatomical location of MCC, period of diagnosis, deprivation status, and risk of non‐melanoma skin cancers (NMSCs) were examined relative to gender and compared in OTR with non‐transplanted patients (Table 1). Chi‐Square tests were used to compare differences in co‐variates by sex and transplant status (Tables 1 and 2). Deprivation was measured based on electoral division (ED) of residence. Patients were assigned a deprivation score on the basis of their Electoral District of residence. The index of deprivation used is based on information collected on education, unemployment and other socioeconomic factors. The level of deprivation in each ED was measured and the population was split into quintiles with the 20% least deprived in quintile 1 and the 20% most deprived in quintile 5. Age‐standardised incidence rates were calculated using the 1976 European Standard Population and the standardized incidence ratio (SIR) was calculated for MCC in OTR as compared to the general population, as previously described.^8^

**TABLE 1:** Patient co‐variates of interest and outcomes relative to gender

*n* (%)	Males *n* = 145	Females *n* = 169	Chi‐square *p*‐value
Age groups (years)			
<65	23 (15.9)	12 (7.1)	‐
65–79	60 (41.4)	60 (35.5)	‐
80+	62 (42.8)	97 (57.4)	0.009
Average age at diagnosis (years)	75.07	79.69	‐
NMSC			
No history of NMSC	72 (49.7)	116 (68.6)	‐
1 NMSC	45 (31.0)	40 (23.7)	‐
2 NMSC	27 (18.6)	13 (7.7)	‐
3 NMSC	1 (0.7)	0 (0.0)	0.002
Deprivation			
1 – Least deprived	22 (15.2)	35 (20.7)	‐
2	13 (9.0)	16 (9.5)	‐
3	24 (16.5)	17 (10.1)	‐
4	23 (15.9)	30 (17.7)	‐
5 – Most deprived	52 (35.9)	62 (36.7)	‐
Missing	11 (7.6)	9 (5.3)	0.458
Overall survival at 5 years			
Alive	65 (44.8)	64 (37.9)	‐
Died from MCC	45 (31.0)	52 (30.8)	‐
Died from other cause	35 (24.1)	53 (31.4)	0.305
Period of diagnosis			
1994–2003	44 (30.3)	59 (34.9)	‐
2004–2014	101 (69.7)	110 (65.1)	0.390

Abbreviations: MCC, Merkel cell carcinoma; NMSC, non‐melanoma skin cancer.

**TABLE 2:** Patient co‐variates of interest and outcomes relative to transplant status

*n* (%)	MCC in the general population	MCC in OTR	Fisher's exact *p*‐value
	*n* = 304	*n* = 10	
Gender			
Male	135 (44.4)	10 (100)	‐
Female	169 (55.6)	0 (0)	<0.001
Age groups (years)			
<65	31 (10.2)	4 (40)	‐
65–79	114 (37.5)	6 (60)	‐
80+	159 (52.3)	0 (0)	<0.001
Location of MCC			
Head and neck	163 (53.6)	7 (70)	‐
Trunk	11 (3.6)	1 (10)	‐
Upper limb	36 (11.8)	1 (10)	‐
Lower limb	68 (22.4)	0 (0)	‐
Other	26 (8.5)	1 (10)	0.237
NMSC			
No history of NMSC	188 (61.8)	0 (0)	‐
1 NMSC	82 (27.0)	3 (30)	‐
2 NMSC	33 (10.9)	7 (70)	‐
3 NMSC	1 (0.3)	0 (0)	<0.001
Deprivation			
1 – Least deprived	57 (18.8)	0 (0)	‐
2	29 (9.5)	0 (0)	‐
3	40 (13.2)	1 (10)	‐
4	51 (16.8)	2 (20)	‐
5 – Most deprived	107 (35.2)	7 (70)	‐
Missing	20 (6.6)	0 (0)	0.335
Overall survival at 5 years			
Alive	126 (41.4)	3 (30)	‐
Died from MCC	90 (29.6)	7 (70)	‐
Died from other cause	88 (28.9)	0 (0)	0.013
Period of diagnosis			
1994–2003	100 (32.9)	3 (30)	‐
2004–2014	204 (67.1)	7 (70)	1.000

Abbreviations: MCC, Merkel cell carcinoma; NMSC, non‐melanoma skin cancer; OTR, organ transplantation recipient.

Competing risks analysis was performed with patients recorded as either alive, having died from MCC or from other causes 5‐year‐post diagnosis. Death due to MCC was the event of interest and death due to other causes was the competing risk. Fine‐Grey competing risks regression models were used to test whether gender was associated with the hazard of dying from MCC. All covariates of interest were included in the initial model. Gender was included in the final models, which were adjusted for the covariates of interest, with variables significant at 5% included in the final model. The proportional hazards assumption was tested by examining whether the interactions between survival time and the co‐variates were significant. This was done by the inclusion of time varying co‐variates in the models.

STATA SE (version 15.1 StataCorp) was used for the data analysis.

## 3 RESULTS

**FIGURE 1:**
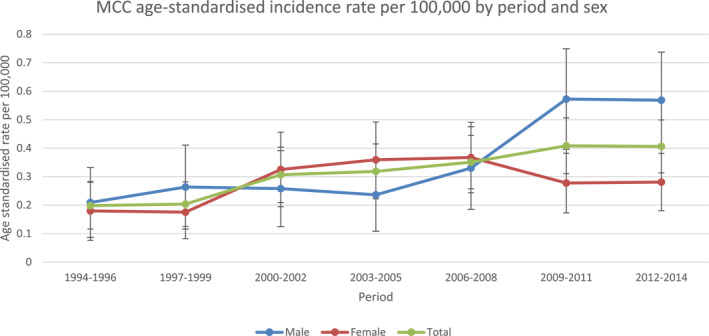
Age standardized incidence rate

In total 314 cases of MCC were identified between 1994 and 2014. Comparison between age‐standardised rates between the earliest period (1994–1996) with the latest period (2012–2014) showed an increase of 105% in total (Figure 1). Age‐standardised rates for the population increased significantly (*p*‐value = 0.0004). When comparing the trends between males and females, there was no significant difference. Over the 20‐year period there were greater numbers of females than males diagnosed with MCC (females, *n* = 169 [53.8%], males *n* = 145 [46.2%]). The average age at diagnosis was 77.6 years (males 75.1 years vs. females 79.7 years) (Table 1). Male patients were more likely to present under the age of 65 years (males *n* = 23 [15.9%] vs. females *n* = 12 [7.1%]). While female patients were more likely to present over 80 years of age (females *n* = 97 [57.3%] vs. males *n* = 62 [42.8%]). One patient developed MCC under the age of 20 years, with two patients diagnosed in the third decade of life and 2 further patients diagnosed in their fourth decade. Of note, no patient was diagnosed with more than one MCC.

**FIGURE 2:**
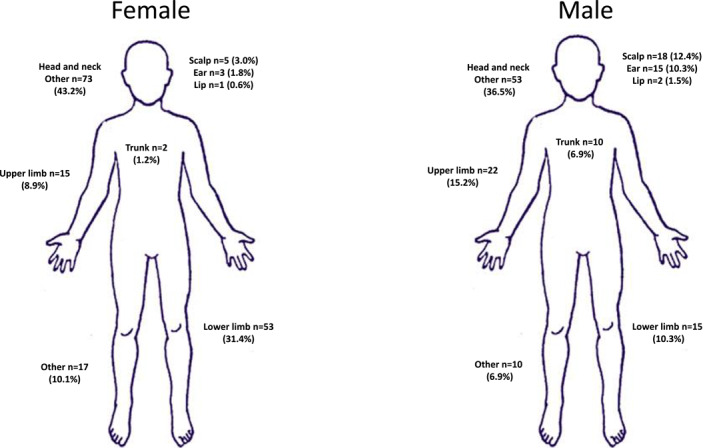
Variation in anatomical location of Merkel cell carcinoma by gender

Differences in anatomical location of MCCs were noted between genders. Overall, the majority of MCC cases presented on the head and neck (*n* = 170, 54.1%). Males were more likely to present with MCC in this location (males *n* = 190 [60.7%] vs. females *n* = 152 [48.5%]). The lower limb was the second most common anatomical site for MCC development (*n* = 68, 21.7%). Almost three times as many females developed MCC in this site compared with their male counterparts (Figure 2).

In total, 188 patients (59.9%) diagnosed with MCC had no history of NMSC. While 126 patients (40.1%) had a history of NMSCs (males *n* = 73 [57.9%], females *n* = 53 [42.1%] [*p*‐value is 0.002]). Malignant melanoma was diagnosed in 4 (1.3%) of MCC patients. MCC was commonest in the two most deprived groups, *n* = 167 (53.2%), compared with 86 cases (27.4%) occurring in the two least deprived group. For both males and females, over half of cases occurred in the two most deprived groups (males *n* = 75, 51.7% vs. females *n* = 92, 54.4%).

At the censoring date (31 December 2014), 106 (33.6%) patients were alive. Two hundred and eight patients had died, 100 (31.8%) died from MCC. Of these, 46 were male and 54 were female. Of those who died from MCC, average survival time was 3.5 years (males 3.4 years, females 3.5 years). Of those diagnosed with MCC on the head & neck, 35% died of their disease, while 44% of those with MCC in other anatomical sites died of MCC.

Ten MCC cases were identified in OTR (nine renal transplant recipients, one heart transplant recipient) (Table 2). There were 3346 solid organ transplants recorded in the Irish national transplant registries over the period included in this study.^9^ In keeping with overall changes MCC case number, the number of MCC cases diagnosed in OTR more than doubled between the first and second periods examined in this study. The SIR for MCC in OTR was 59.96 (C.I. 19.3, 139). All OTR diagnosed with MCC were male. The average age at diagnosis in OTR was 65.1 years compared to 79.0 years in the non‐transplanted patients. The average time from transplantation to the development of MCC was 14.1 years. In OTR, the majority of MCCs developed on the head and neck (*n* = 7,70%). All of the OTRs in this study also had been diagnosed with an NMSC, with (*n* = 7, 70%) of OTR having had two or more NMSCs. All but one of the MCC cases in OTR (*n* = 9, 90%) were diagnosed in the two most deprived groups. Of all OTR diagnosed with MCC, the majority (*n* = 7, 70%) died from this disease. Median survival for OTR who died from MCC was 0.14 years.

Two‐hundred‐fifty‐one patients (79.9%) underwent surgical intervention, 106 patients (33.8%) received radiation treatment while 28 patients (8.9%) received chemotherapy.

In competing risks regression, gender was not significantly associated with the risk of dying from MCC, with females having a non‐significantly higher hazard of dying from MCC. OTR and less deprived patients were at significantly increased risk of dying from MCC (Table 3). The proportional hazards assumption was not violated in the cause specific survival analysis.

**TABLE 3:** Competing risks regression analysis up to 5 years post diagnosis for MCC deaths

	SHR	*p*‐value	95% LL	95% UL	Wald test *p*‐value
Gender					
M	1	‐	‐	‐	‐
F	1.05	0.830	0.68	1.61	0.830
Patients					
Patients without organ transplant	1	‐	‐	‐	‐
OTR patients	4.97	0.002	1.78	13.83	0.002
Deprivation					
1 – Least deprived	1	‐	‐	‐	‐
2	1.61	0.201	0.78	3.32	‐
3	0.84	0.627	0.41	1.72	‐
4	0.48	0.049	0.24	1.00	‐
5 – Most deprived	0.66	0.153	0.37	1.17	‐
Missing	0.48	0.159	0.17	1.34	0.033

Abbreviations: LL, lower limit; MCC, Merkel cell carcinoma; OTR, organ transplantation recipient; SHR, sub hazard ratio; UL, upper limit.

## 4 DISCUSSION

This study which examined national registry data over 2 decades, supports epidemiological trends around increasing MCC incidence but also identified some gender related patterns that warrant further investigation. We demonstrated more than a 100% increase in the number of MCC cases diagnosed in Ireland between 2004 and 2014 compared with the previous decade (Table 1). The age standardized incidence rate trended significantly upwards over the period. As the ICD‐10 code for MCC was first coined in 2009, and CK‐20 staining for MCC became available in 1995, the years reviewed in this paper reflect a gradual transition to more accurate registration of cases. Similarly, between 2000 and 2013, a 95% increase in MCC incidence was noted in the United States, higher than increases seen in other UV associated skin malignancies, such as melanoma (57% increase).^2^ It has been suggested that the rapid increase in incidence rates for melanoma may be in part explained by increased diagnostic scrutiny and overdiagnosis.^10^ MCC, however, does not have a benign counterpart or precursor lesion. Confirmatory immunohistochemistry also allows for greater pathological diagnostic certainty.

Of note in this Irish, largely fair‐skinned homogenous population, increasing incidence of MCC over the periods examined was seen particularly in older patients and in men, with a less substantial increase in female patients (Figure 1). MCC is known to be largely a disease of the elderly. Indeed, in this cohort of patients the average age at diagnosis was 77.6 years of age with <3% of cases diagnosed in patients under 50 years of age.

MCC is rare, typically described as occurring in fair‐skinned, elderly male patients on sun exposed skin, but in our study a female preponderance (53.8%) in MCC was noted. While MCC is traditionally thought be commoner in males,^11^, ^12^, ^13^, ^14^, ^15^ there are regional variations with a number of reviews of MCC in Northern Europe also demonstrating a female predominance.^16^, ^17^, ^18^, ^19^, ^20^, ^21^ This incidence varied with age, with males more likely to develop MCC under the age of 65 years, while females were more likely to present over 80 years of age. A review from Queensland, Australia revealed the highest incidence rate of MCC internationally (1.6 per 100 000),^12^ supporting the link between high rates of UV exposure and development of MCC. The anatomical location of MCC development in this review supports this concept. Overall, the head and neck was the most common site. MCC developed more commonly on the ears and scalp in males where there is likely to be less hair cover. Forty percentage of MCC patients in the Irish cohort also had a history of NMSCs. There is a significant difference in NMSC history by sex, with females less likely to have NMSC history. Polyoma virus negative MCCs are shown more likely to occur in sun exposed regions, and have a higher mutational burden including UV light signature mutations.^22^ Poor survival rates from MCC have been demonstrated in New Zealand which is thought be linked to high UV exposure, fair skin type and lower rates of MCPyV positivity in this region.^23^ Interestingly, the lower limbs were noted overall to be the second most common location for MCC development. MCC numbers in this location were three times more common in females. While squamous cell carcinoma in situ and melanoma are recognized as commonly occurring on the lower limbs of females, a propensity of MCC for this site has not previously been described.

Although female MCC patients presented at an older age, likely with an increased burden of co‐morbidities, they had longer average survival times. A recent study demonstrated improved survival in females with MCC.^6^ The authors suggest this may be due to inherent immunological differences between genders. In competing risks regression in this study, gender was not statistically significantly associated with the risk of dying from MCC, with females having a non‐significantly higher hazard of dying from MCC.

The NCRI has demonstrated higher overall cancer incidence in the most deprived patients in Ireland compared with the least deprived.^24^ The opposite pattern has been observed in both MCC and malignant melanoma.^24^, ^25^ This study demonstrated that patients from a less deprived socioeconomic group were at increased risk of dying from MCC. Other studies have noted patients from lower socioeconomic groups tend to have higher rates of MCC in the head and neck region and advanced disease^26^ and may have poorer survival rates than patients with MCC in other sites.^27^ This may be in part have been related to more conservative surgical approaches and a higher propensity for metastatic spread. This is in contrast to our review, in which a lower percentage of patients with MCC located on the head and neck died of their disease, compared with cases of MCC located on other anatomical locations. In this review a high proportion (90%) of OTR with MCC were in the two most deprived groups. It has been shown however that deprivation in itself is associated with a poor prognosis in renal disease.^28^

Our group recently documented increasing MCC numbers in renal transplant recipients as the mean age at transplantation increases^29^ Our findings paralleled other studies that have shown skin cancers occur an average of 30 years earlier in OTR than those in the general population.^30^ The almost 60‐fold increase in relative risk for MCC within our OTR population is even greater than that shown in a review of 189 498 solid OTR in the United States demonstrating an overall 23.8‐fold increased risk for MCC. The authors suggest that immunosuppressive medications may act synergistically with UV radiation to increase MCC risk^31^ and potentiate metastatic spread as indicated in the very short median survival time of 0.225 years for OTR cohort who died from MCC.

There are a number of limitations regarding this analysis. The number of OTRs in this cohort is proportionally small when compared with the general population. The NCRI does not record subtype of keratinocyte cancer as a cause of death. While this is a limitation of this study, it is assumed, those diagnosed with MCC died from this malignancy rather than another NMSC. Polyoma virus status was not available on the cases in this review.

This population based study provides epidemiological, clinical and outcome data for MCC over a 20‐year period. Females were shown to develop MCC at an older age, more frequently on sites less typically sun exposed, are less likely to have had prior NMSCs, and have a longer survival time than their male counterparts. In contrast, male patients with MCC were on average younger than female counterparts with MCC more likely to occur on the head and neck and in association with a prior history of NMSC. Further studies correlating MCPyV status and UV mutational load of tumours in these different groups are required. The role of a functioning immune system in preventing MCC was highlighted in our cohort of OTR who had a significantly increased incidence of MCC in male OTR, with an earlier age at presentation, association with other NMSC and significantly reduced median survival.

## CONFLICTS OF INTEREST

The authors have no conflicts of interest to declare.

## AUTHOR CONTRIBUTIONS

**E. Keeling:** Conceptualization; Data curation; Formal analysis; Investigation; Methodology; Project administration; Writing – original draft; Writing – review & editing. **E. O'Leary:** Data curation; Formal analysis; Investigation; Methodology; Writing – review & editing. **S. Deady:** Data curation; Formal analysis; Investigation; Methodology. **J. P. O Neill:** Formal analysis; Investigation; Supervision; Writing – review & editing. **P. J. Conlon:** Conceptualization; Data curation; Formal analysis; Investigation; Supervision; Writing – review & editing. **F. J. Moloney:** Conceptualization; Data curation; Formal analysis; Investigation; Methodology; Supervision; Writing – review & editing.

## Data Availability

Data sharing is not applicable to this article as no new data were created or analyzed in this study.
